# Controlled water vapor transmission rate promotes wound-healing via wound re-epithelialization and contraction enhancement

**DOI:** 10.1038/srep24596

**Published:** 2016-04-18

**Authors:** Rui Xu, Hesheng Xia, Weifeng He, Zhichao Li, Jian Zhao, Bo Liu, Yuzhen Wang, Qiang Lei, Yi Kong, Yang Bai, Zhihui Yao, Rongshuai Yan, Haisheng Li, Rixing Zhan, Sisi Yang, Gaoxing Luo, Jun Wu

**Affiliations:** 1 Institute of Burn Research, Southwest Hospital; State Key Lab of Trauma, Burn and Combined Injury; Chongqing Key Laboratory for Disease Proteomics, Third Military Medical University, Chongqing 400038, China; 2 State Key Laboratory of Polymer Materials Engineering, Polymer Research Institute of Sichuan University, Chengdu 610065, China

## Abstract

A desirable microenvironment is essential for wound healing, in which an ideal moisture content is one of the most important factors. The fundamental function and requirement for wound dressings is to keep the wound at an optimal moisture. Here, we prepared serial polyurethane (PU) membrane dressings with graded water vapor transmission rates (WVTRs), and the optimal WVTR of the dressing for wound healing was identified by both *in vitro* and *in vivo* studies. It was found that the dressing with a WVTR of 2028.3 ± 237.8 g/m^2^·24 h was able to maintain an optimal moisture content for the proliferation and regular function of epidermal cells and fibroblasts in a three-dimensional culture model. Moreover, the dressing with this optimal WTVR was found to be able to promote wound healing in a mouse skin wound model. Our finds may be helpful in the design of wound dressing for wound regeneration in the future.

The skin is the largest organ in the human body and plays an important role in maintaining body fluid, electrolytes and nutritional components. For patients suffering from severe acute or chronic wounds, such as burns or other extensive skin loss, this barrier has been destroyed.

Wound healing is a complicated pathophysiological process that requires a desirable microenvironment, in which moisture is one of the most important factors^1^,^2^. All cells of the human body live in their own fluid microenvironment. After injury, the evaporative water loss from the wound surface can be approximately twenty times greater than that of normal skin^3^,^4^. When the wound is directly exposed to air, it dehydrates, and a scab is formed, which aims in protecting the wound from bacterial infection. However, it is clear that a cell in a dry or low moisture microenvironment will lose its vitality and function and even die. It has also been reported that healing under wet conditions is faster than dry scab conditions^1^,^2^,^5^,^6^,^7^. Thus, a suitable wound dressing is essential in controlling water evaporation from a wound. The primary fundamental function and requirement for a wound dressing is to maintain the optimal moisture for wound healing.

Great progress in wound dressings has been made in the past quarter century, but the current studies primarily focus on investigating the effects of different materials on wound healing, using specific cells for optimal regeneration or trying to encapsulate chemical drugs or biomolecules to accelerate wound healing^8^,^9^,^10^,^11^,^12^,^13^. The basic physical property of wound dressings that may influence the wound healing process has not yet been studied thoroughly, i.e. the water vapor transmission rate (WVTR), which directly regulates the moisture microenvironment of wound healing.

The ability of a dressing to control water loss can be determined by the WVTR. Therefore, the wound surface moisture can be regulated through the use of various wound dressings with different WVTRs. An extremely high WVTR may lead to the dehydration of a wound, whereas an unacceptably low WVTR may cause the accumulation of wound exudates. Hence, a dressing with a suitable WVTR is required to provide a moist environment for establishing the best milieu for natural healing.

Queen *et al*. reported that a certain range of WVTRs could support adequate moisture conditions for wound healing without dehydration^3^. However, this level was suggested indirectly based on half of the evaporative water loss of a granulating wound, and no detailed data from animal experiments were presented. Schunck’s study demonstrated that a water vapor-permeable wound dressing was conducive to wound healing^14^. However, the WVTR of the dressing used in their study was not tested, and they did not provide a recommended WVTR range.

In this study, the moisture content of wounds was regulated by a series of polyurethane (PU) membrane dressings with different WVTRs. The optimal WTVR of a dressing for wound healing was determined using both *in vitro* and *in vivo* studies. We determined that the dressing with a WVTR of approximately 2028.3 g/m^2^·24 h could maintain the optimal moisture content for the proliferation and function of epidermal cells and fibroblasts in a three-dimensional culture model. Moreover, the optimal WVTR was confirmed through the observation of the wound healing, granulation tissue growth and reepithelialization in a mouse skin wound model. Furthermore, wound dressings with different WTVRs were observed to influence wound healing by altering the expressions of α-smooth muscle actin (α-SMA), proliferating cell nuclear antigen (PCNA) and E-cadherin as well as the production of epithelial growth factor (EGF), which resulted in changes in the function, proliferation and migration of fibroblasts and epidermal cells. To the best of our knowledge, this is the first study to demonstrate that a dressing with a WVTR of approximately 2028.3 g/m^2^·24 h is optimal and necessary for wound healing, and this data may be helpful in the design of wound dressing for wound regeneration in the future.

## Results

### Microporous PU membranes with graded WVTRs

**Figure 1:**
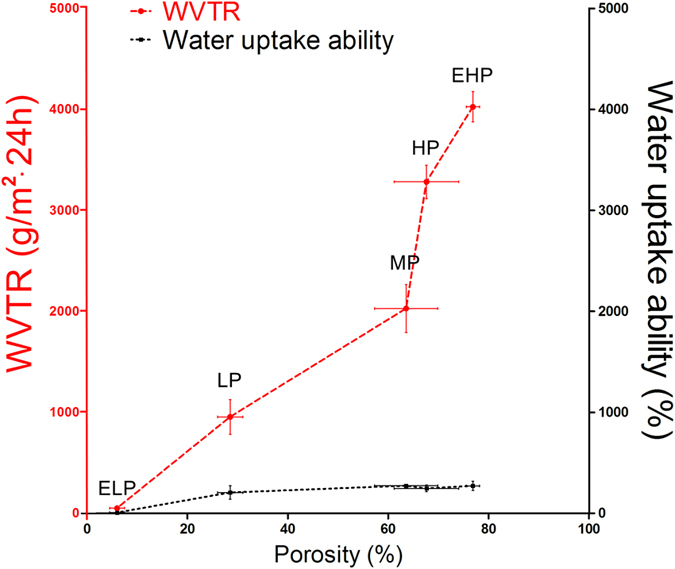
WVTR, water uptake ability and porosity of the prepared PU membranes. The values were calculated as the mean ± SD (n = 3).

**Figure 2:**
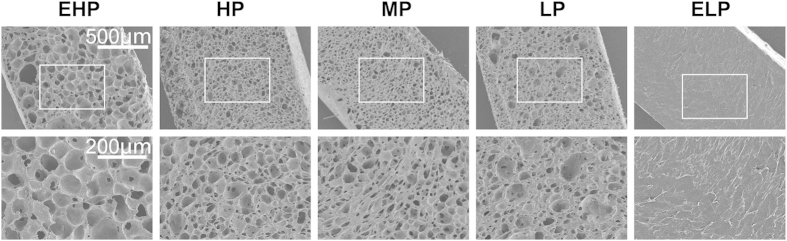
SEM fractographs of the PU membranes showing their porous structure. The porosity of PU membrane could be regulated by changing the PU/DMF/sodium citrate ratio.

In this study, microporous PU membranes with graded WVTRs were prepared by changing the porosity of the membrane (Figs 1 and 2). The PU/n,n-dimethylformamide (DMF)/sodium citrate ratios of 25 g/200 ml/75 g, 25 g/200 ml/55 g, 25 g/200 ml/45 g and 40 g/200 ml/40 g corresponded to membrane average porosities of 76.9%, 67.6%, 63.6% and 28.5%, respectively. Meanwhile, the corresponding average WVTRs of the membranes were 4025.8 (extremely high permeability, EHP), 3282.0 (high permeability, HP), 2028.3 (medium permeability, MP) and 954.8 g/m^2^·24 h (low permeability, LP), respectively (Fig. 1). The WVTR of the pure PU membrane was 50.2 g/m^2^·24 h (extremely low permeability, ELP; Fig. 1). The water uptake abilities of the membranes were very similar, with the exception of the ELP-PU membrane, which was made of pure PU.

### Effects of moisture regulated by PU membranes with different WVTRs on the proliferation and function of epidermal cells and fibroblasts in a three-dimensional culture model

We examined the ability of different PU membranes to control water loss using an *in vitro* three-dimensional culture model. Additionally, the influence of the water loss of the model on the gel contraction and cell proliferation were investigated.

**Figure 3:**
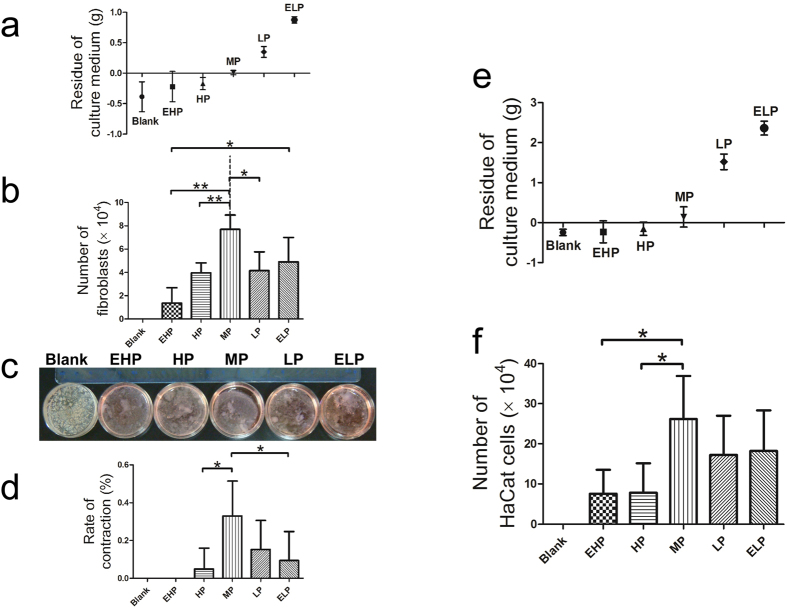
The MP-PU membrane controlled water loss at a suitable level which enhanced gel contraction and cell proliferation. (**a**) Residue of the culture medium in the FPCL model, (**b**) number of fibroblasts, (**c**) FPCL models for the collagen contraction assay and (**d**) rate of contraction. The residue of culture medium in the MP-PU group was 0.01 g, and its rate of contraction was larger than that of the HP and ELP groups. The values were calculated as the mean ± SD (n = 5), **p < 0.01, *p < 0.05. (**e**) Residue of the culture medium in the HaCat three-dimensional culture model and (**f**) the number of HaCat cells. Cell numbers in the MP group were greater than those in the EHP and the HP groups. The values were calculated as the mean ± SD (n = 5), *p < 0.05.

In the fibroblast three-dimensional culture model, i.e. fibroblast populated collagen lattice (FPCL) model, it was observed that the culture medium in the blank and EHP groups evaporated completely (the culture medium and the gel completely dried out). In contrast, large volumes of culture medium remained in the LP and ELP groups (Fig. 3c). The average culture medium residue was measured to be 0.01 g when the culture dish was covered with MP-PU, and the average rate of contraction was 32.9%, which was larger than those of the HP and ELP groups (4.9% and 9.4%, respectively; Fig. 3a,d). It was also observed that the number of fibroblasts in the MP group was greater than that in the EHP, HP and LP groups (Fig. 3b).

In the HaCat cell three-dimensional culture model, we also determined that MP-PU, LP-PU and ELP-PU could control water loss, and cell number in the MP group was greater than that in the EHP and HP groups (Fig. 3e,f).

### Medium-permeability PU membrane significantly accelerated wound healing compared with other groups

**Figure 4:**
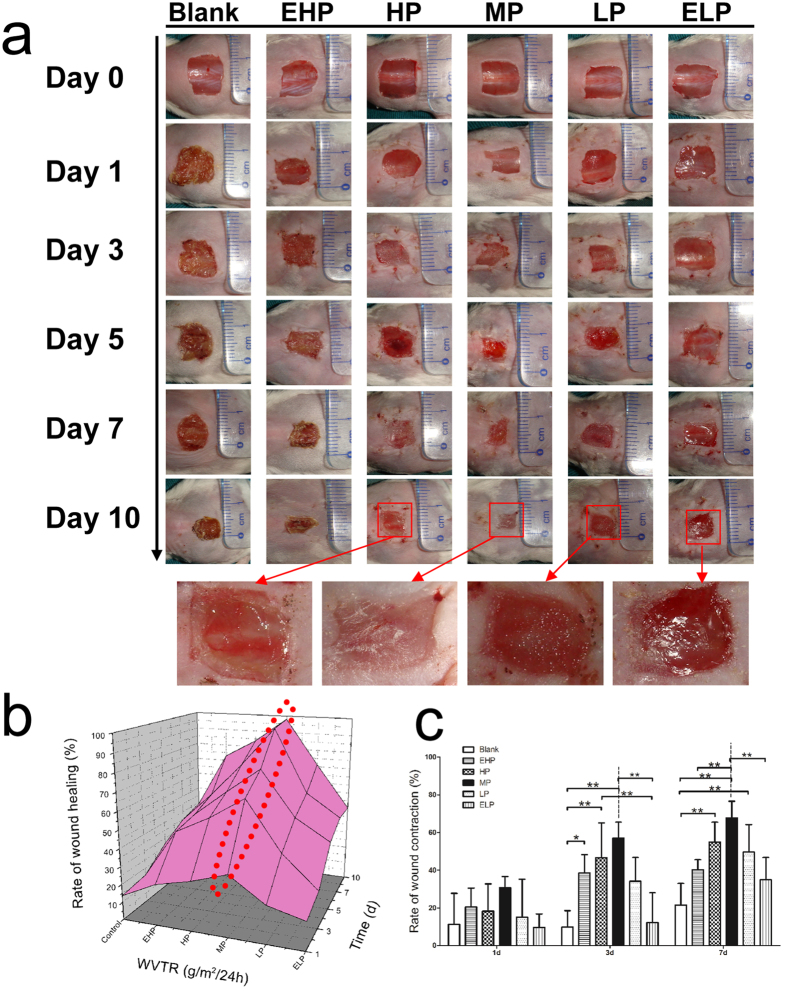
Wound healing experiment. (**a**) The macroscopic appearance of the wounds postsurgery in the six groups at different time-points. (**b**) A three-dimensional diagram of wound healing. (**c**) The rates of wound contraction at different times. The values were calculated as the mean ± SD (n = 5), **p < 0.01, *p < 0.05. Wound healing was observed to accelerate when the MP-PU was applied.

In the mouse wound healing model, wound healing in the MP group was enhanced compared with that in the blank, EHP, HP, LP and ELP groups. As shown in Fig. 4a, the macroscopic appearance of wounds at different times post-wounding were quite different. Wounds in the blank and EHP groups were dehydrated, and scabs formed. Conversely, exudates were observed in the LP and ELP groups. In contrast, the wounds in the MP and HP groups appeared to be clean and moist, especially in the MP group.

Meanwhile, the rate of wound healing at different time intervals was determined. Based on the three-dimensional diagram of wound healing (Fig. 4b), it was clear that the rate of wound healing in the MP group was larger than that in the other groups. At day 7, a reduction of approximately 95.6% wound size was observed in the MP group, whereas the wound size reduction in blank, EHP, HP, LP and ELP groups were 34.8%, 53.2%, 73.4%, 59.0% and 46.0%, respectively. Hence, the application of the PU membrane with medium permeability significantly accelerated wound healing compared with the other groups, especially on days 5, 7 and 10 postsurgery (Supplementary Fig. S2).

### MP-PU membrane significantly increased wound contraction by promoting granulation formation and α-SMA expression

Rapid wound closure might result from wound contraction or/and re-epithelialization. Firstly, the rate of wound contraction was determined in the mouse wound healing model, and it was similar at day 1. However, wound contraction was significantly enhanced by treatment with the MP-PU membrane in comparison to the wound contraction in the blank and the ELP groups at day 3. It was also observed that the rate of contraction in the MP group was significantly higher than that in the blank, EHP and ELP groups at day 7 (Fig. 4c). And on the 7^th^ day, the average rates of wound contraction of blank, EHP, HP, MP, LP and ELP groups were 21.4%, 40.2%, 54.8%, 67.7%, 49.6% and 34.8%, respectively.

**Figure 5:**
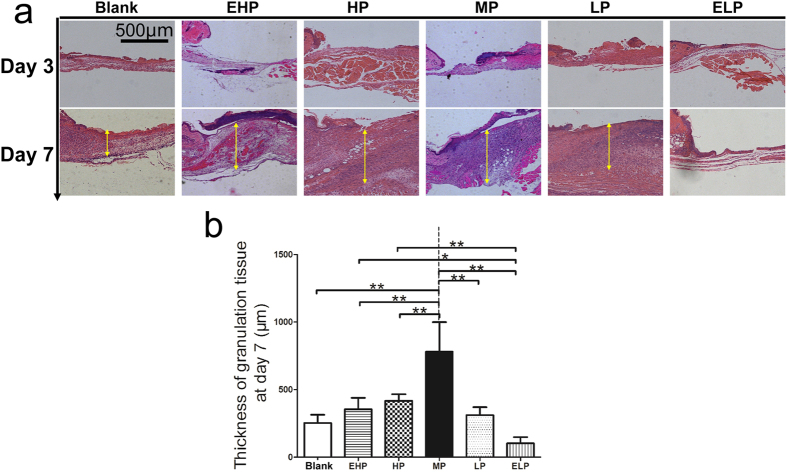
Formation of granulation tissue. (**a**) H&E staining of the wound tissue at 3 and 7 days post-wounding and (**b**) Thickness of granulation tissue. The average thickness of granulation tissue in the MP group was much thicker than that in other groups. The values were calculated as the mean ± SD (n = 5), **p < 0.01, *p < 0.05.

To understand the possible mechanism of the enhanced wound contraction, firstly, HE staining of the wound tissue was performed to evaluate the amount of the newly regenerated granulation tissue (Fig. 5a). As presented in Fig. 5b, on the 7^th^ day, the average thicknesses of the wound granulation tissue covered with the MP-PU membrane was 780.1 μm, which was much thicker than that in blank, EHP, HP, LP and ELP groups (252.7 μm, 353.5 μm, 416.2 μm, 310.9 μm and 102.4 μm, respectively; Fig. 5b).

**Figure 6:**
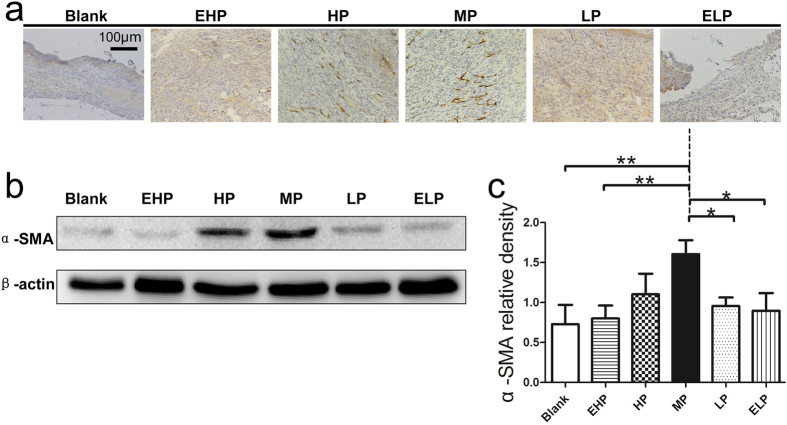
Expression of α-SMA in the wound tissue. (**a**) α-SMA immunohistochemical staining in the wound tissue at 7 days post-surgery. (**b**) α-SMA and β-actin protein levels were determined by Western blot, and (**c**) relative densities of α-SMA protein level in each group are shown. The values were calculated as the mean ± SD (n = 3), **p < 0.01, *p < 0.05.

Secondly, α-SMA expression in wound tissue was examined by both immunohistochemical staining (Fig. 6a) and western blotting (Fig. 6b). It was revealed that α-SMA expression was significantly enhanced in MP group compared to blank, EHP, LP and ELP groups at the protein level (Fig. 6c).

### MP-PU membrane promoted re-epithelialization by augmenting the proliferation and the migration of keratinocyte

**Figure 7:**
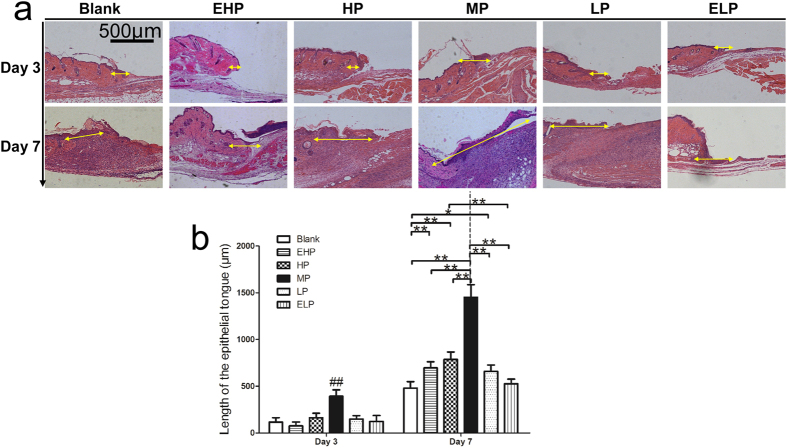
Re-epithelialization of the wound. (**a**) The newly formed epithelium in the wound tissue at 3 and 7 days post-wounding and (**b**) the length of the newly formed epithelium was significantly increased when MP-PU membrane was applied. The values were calculated as the mean ± SD (n = 5), **p < 0.01, *p < 0.05.

The length of the neo-epithelium was measured based on the HE staining sections. At days 3 and 7, histological analysis revealed that the average length of the neo-epithelium in MP group was 392.4 μm and 1450.6 μm, respectively; these values were significantly greater than those observed for the blank, EHP, HP, LP and ELP groups (Fig. 7).

**Figure 8:**
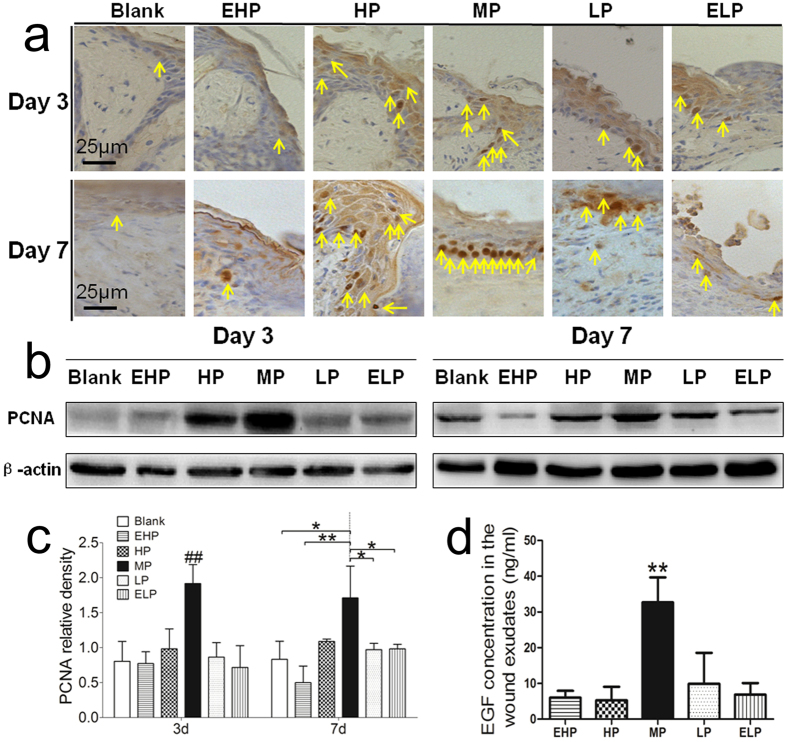
Proliferation of keratinocytes in the wound. (**a**) PCNA immunohistochemical staining in the wound tissue at day 3 and day 7 post-surgery. (**b**) PCNA and β-actin protein levels were determined by Western blot, and (**c**) relative densities of PCNA protein level in each group are shown. The values were calculated as the mean ± SD (n = 3), **p < 0.01, *p < 0.05. (**d**) EGF concentration in the wound exudates. The concentration of EGF in the MP group was much higher than that in other groups. The values were calculated as the mean ± SD (n = 3), **p < 0.01.

Enhanced re-epithelialization might result from the keratinocyte proliferation or the keratinocyte migration. Thus, we first investigated the keratinocyte proliferation at the wound edge. PCNA expression in the epidermis was detected by immunohistochemical staining (Fig. 8a), and it was observed that the intensity of PCNA staining at the wound edge in MP group was higher than that in the other groups at day 3. Western blot results also indicated that the protein level of PCNA in the MP group was significantly higher than that in the other groups. At day 7, we still observed that PCNA expression in the MP group was significantly increased compared with the blank, EHP, LP and ELP groups (Fig. 8b,c). Meanwhile, the EGF concentration of the wound exudates was determined, and it was observed that the concentration of EGF in the MP group was much higher than that of the other groups (Fig. 8d).

**Figure 9:**
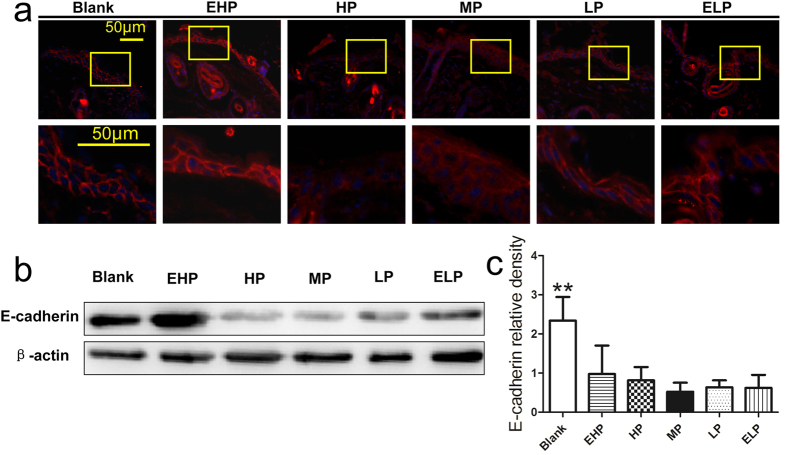
Migration of keratinocytes in the wound. (**a**) The expression of E-cadherin at the wound edge at 3 days post-wounding, which revealed that the staining of E-cadherin at the wound edge decreased without a typical linear pattern when MP-PU membrane was applied. (**b**) E-cadherin and β-actin protein levels were determined by Western blot, and (**c**) relative densities of E-cadherin protein level in each group are shown. The values were calculated as the mean ± SD (n = 3), **p < 0.01.

Second, we investigated the migration of keratinocyte in the neo-epithelium at the wound edge. As one of the components of adherens junctions, downregulation of E-cadherin might lead to a loosening of cell-cell contact, which contributes to keratinocyte migration^15^. The expression of E-cadherin was detected by immunofluorescence and we observed that the staining of E-cadherin in blank and EHP groups exhibited a typical linear pattern outlining the cell. However, the staining of E-cadherin in other groups was weak and blurred especially in the HP and MP groups (Fig. 9a), which indicated that expression of E-cadherin was down regulated and thus led to the keratinocyte migration at the wound edge. Western blot analysis also revealed that expression of the E-cadherin in the blank group was significantly higher than that in the other groups (Fig. 9b,c, the relative density of E-cadherin in blank group was larger than that of all the others).

## Discussion

Moist wound healing was invented by George Winter in 1962. Although this term is frequently used, it is still not well understood, even after five decades^1^,^16^. Based on this theory, one of the major problems for patients suffering from serious skin defects is total wound surface dehydration^3^. Excessive fluid retention at the wound surface, however, can also result in poor healing and maceration of the surrounding tissue. Therefore, a delicate moisture balance is required for optimal wound healing^17^. Although the ideal moisture required for wound healing is not known clearly and wound surface moisture cannot be determined precisely, the moisture can be regulated by different wound dressings with different permeabilities. The rate of water vapor transmission is an important parameter of the dressing and represents the dressing’s ability to retain moisture. Thus, the effects of different WVTRs on wound healing should be studied, and a suitable WVTR level should be recommended for the design of wound dressings. However, a systematic and detailed evaluation of the bio-function of WVTR in the wound healing process is lacking in most of the current studies.

In this study, microporous PU membranes with graded WVTRs were prepared by changing the porosity of the membrane (Figs 1 and 2). In our study, the oxygen transmission abilities of the membranes were also measured and it was found that permeability coefficients of different membranes were similar (Supplementary Fig. S3), which indicated that WVTR was the main factor that affected wound healing in this study.

First, we observed that the application of the MP-PU membrane controlled water loss at a suitable level and contracted collagen more extensively than did HP-PU and ELP-PU membranes in an *in vitro* three-dimensional culture model, i.e., FPCL model (Fig. 3a,c,d). Secondly HaCat cells instead of fibroblasts were seeded in the three-dimensional culture model, and we observed that the number of cells in the MP group was greater than those observed in the EHP and HP groups (Fig. 3e,f), which means that the proliferation of HaCat cells was enhanced when MP-PU membrane was applied. Based on these results, we hypothesized that application of MP-PU membranes (WVTR: 2028.3 ± 237.8 g/m^2^·24 h) could maintain a suitable moist environment in the wound that could enhance the wound contraction and tissue regeneration, thereby accelerating wound healing.

To investigate the effects of the PU membranes with different WVTRs on wound healing, a full-thickness wound model was established. The *in vivo* test showed that the rate of wound healing significantly increased under the conditions of the MP-PU membrane, especially on days 5, 7 and 10 postsurgery (Fig. 4b and Supplementary Fig. S2).

The rapid wound closure might result from wound contraction or/and re-epithelialization: the two factors were assayed in our animal model. The results revealed that wound contraction was significantly enhanced by treatment with the MP-PU membrane in comparison to the wound contraction in the blank and the ELP groups at day 3. It was also observed that the rate of contraction in the MP group was significantly higher than that in the blank, EHP and ELP groups at day 7 (Fig. 4c). Wound contraction was important for wound closure, especially for animals with loose skin (mouse, rat). Myofibroblasts are one of the main components of granulation tissue and could contract the granulation tissue and pull the edges of wound closer to each other. Thus, myofibroblasts and granulation tissue were critical to wound contraction^18^,^19^,^20^. α-SMA was the marker for myofibroblasts, and the up regulation of α-SMA promoted the differentiation of fibroblasts to myofibroblasts. Meanwhile, the expression of α-SMA could form stress fibers and increase the generation of strong contractile force^20^,^21^. In our research, we observed that the formation of granulation tissue was enhanced and that the expression of α-SMA was elevated in the MP group (Figs 5 and 6); thus, these two factors could explain the enhanced wound contraction when the MP-PU membrane was applied.

Re-epithelialization is a common and key stage of all animals during the wound healing process, especially in tight-skinned species (human, porcine). In our study, histological analysis revealed that the average length of the neo-epithelium in the MP group was 392.4 and 1450.6 μm at days 3 and 7 post-wounding, respectively, which was significantly longer than those of the blank, EHP, HP, LP and ELP groups (Fig. 7). Because we observed that re-epithelialization was enhanced, keratinocyte proliferation and keratinocyte migration, the two key parameters for re-epithelialization were further investigated.

PCNA as a marker of cell proliferation was detected by immunohistochemical staining (Fig. 8a) and western blotting (Fig. 8b). It was observed that the protein level of PCNA at the wound edge in the MP group was significant higher than that in the other groups, especially at day 3 postsurgery. The results indicated that cell proliferation and tissue regeneration was enhanced when the MP-PU membrane was dressed on the wound.

As one of the components of adherens junctions, downregulation of E-cadherin might lead to a loosening of cell-cell contact, which contributes to keratinocyte migration^15^, and the expression of E-cadherin was also detected in our study. Although we observed that the influence of the WVTR of the wound dressings on the expression of E-cadherin were not notable, we still demonstrated that E-cadherin was downregulated in the neo-epithelium at the wound edge when wound dressings were applied (Fig. 9). Because a moist environment was able to enhance cell migration^5^,^6^,^7^, we believed that application of the PU membrane could supply a moist environment in the wound, thus leading to the down regulation of E-cadherin compared to the dry scab, which ultimately promoted keratinocyte migration.

Based on the current studies and our own results, we concluded that wound healing in a moist environment occurred more rapidly compared to that in a dry scab^2^. However, the accumulation of the exudates might cause wound maceration, which was also harmful to wound healing. Therefore, a suitably moist environment supplied by the MP-PU membrane was observed to be the best for wound contraction, keratinocyte proliferation and keratinocyte migration, which resulted in the acceleration of wound healing^2^,^5^,^6^,^7^.

In this study, we prepared PU membranes with graded WVTRs and studied the effect of the WVTR of the dressing on wound healing. The MP-PU (WVTR: 2028.3 ± 237.8 g/m^2^·24 h) membrane maintained optimal moisture for the proliferation and functions of the epidermal cells and fibroblasts in a three-dimensional culture model. More importantly, *in vivo* results revealed that the MP-PU membrane could enhance the function, proliferation and migration of the fibroblasts and epidermal cells by up regulating the expression of α-SMA and PCNA, down regulating the expression of E-cadherin, and by increasing the production of EGF. To our knowledge, this is the first time showing that a dressing with a WVTR of approximately 2028.3 g/m^2^·24 h is optimal and necessary for wound healing, and this data may be helpful in the design of wound dressing for wound regeneration in the future. Further studies concerning the possible mechanism of WVTR on cell activation and gene expression should be performed in the future.

## Conclusions

We prepared PU membranes with graded WVTRs successfully using the particulate leaching method. A WVTR of 2028.3 ± 237.8 g/m^2^·24 h was observed to provide an optimal moist environment locally to promote wound healing. The dressing with the optimal WVTR enhanced wound healing by improving the proliferation and function of epidermal cells and fibroblasts.

## Materials and Methods

### Materials and animals

The PU used in this study was a medical-grade product purchased from Lubrizol, USA. Analytical-grade DMF and sodium citrate were obtained from Kelong Chemical Reagent Factory, Chendu, China.

Balb/c neonatal mice and Balb/c mice (male, 18 to 20 g) were purchased from the Experimental Animal Department of the Third Military Medical University. The experiment protocols were approved by the Institutional Animal Care and Use Committee of Third Military Medical University. All of the methods were carried out in accordance with the guidelines of The Third Military Medical University. The animals were individually raised in plastic cages and were adaptively bred for 1 week before the experiments were conducted.

### Preparation of the graded WVTR PU membranes with different porosities

Microporous PU membranes were prepared using the particulate leaching method as previously described^22^. More importantly, we were able to prepare several PU membranes with graded WVTRs as well as successfully determined the proper ratio of PU/DMF/sodium citrate for controlling the membranes’ WVTRs. Briefly, based on our preliminary experiments, a solution of PU/DMF/sodium citrate (25 g/200 ml/75 g, 25 g/200 ml/55 g, 25 g/200 ml/45 g or 40 g/200 ml/40 g, respectively) was mixed thoroughly using a sodium citrate particle size of 75 ~ 150 μm. The solution was then cast in a polytetrafluoroethylene (PTFE) mold with a casting thickness of 1 mm. The PTFE mold was kept in an oven at 100 °C for 4 h to allow the DMF to evaporate. After evaporation, a solid PU membrane containing sodium citrate particles was obtained, and the obtained PU membrane was immersed in deionized water for 72 h (the deionized water was refreshed every day) to extract the sodium citrate particles and solvent residues. Finally, the membrane was dried at 40 °C for 6 h, and the microporous PU membrane was obtained.

Besides, a solution of PU/DMF (25 g/200 ml) was mixed to prepare the pure PU membrane without microporous structure, the other procedures were the same as described above.

### Porosity evaluation

Porosities of the prepared PU membranes were determined as previously reported^23^. The sample was cut into a square shape, and the length, width and height of the sample were measured using a vernier caliper to calculate the volume. The sample was then weighed and subsequently immersed in absolute ethanol. The sample was weighed again after it was saturated. Porosity was calculated as


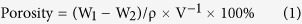


where W_1_ and W_2_ are the weights of the PU membrane before and after immersion in alcohol, respectively. V represents the volume of the sample, and ρ is the density of absolute ethanol (0.79 g/ml).

### WVTR

To determine the moisture permeability of the PU membranes, the WVTR was measured according to the American Society for Testing and Materials (ASTM) standard^24^. Briefly, a sample was cut into a disc and mounted on the mouth of a cylindrical cup containing distilled water. The sample and cup were sealed with Teflon tape across the edge and then placed into a 37 °C incubator at 50% relative humidity. The results were recorded and analyzed automatically by the water vapor transmission rate tester (W3/030, Labthink, China). All measurements were repeated three times (n = 3).

### Water uptake ability

The water uptake abilities of the membranes were determined as previously described^25^. Dry samples were cut into 1 cm × 1 cm square shapes and weighed. Samples were then immersed in the deionized water for 24 h and then weighed after the surplus water on the surface of the membrane was removed using filter paper. The water uptake ability was calculated as follows:


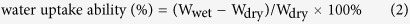


### Effects of moisture regulated by PU membranes with different WVTRs on proliferation and function of epidermal cells and fibroblasts in a three-dimensional culture model

A three-dimensional culture model was established as described previously with several modifications^26^,^27^. Soluble collagen was extracted from rat tails. Fibroblasts were isolated from Balb/c neonatal mice as previously described^9^,^28^. The 3rd-passage subcultured fibroblasts were used to test proliferation ability and function in a three-dimensional culture model. The fibroblast suspension was adjusted to 2 × 10^5^/ml, and 700 μL cell suspension, 100 μL 10× phosphate-buffered saline (PBS; pH 7.4) and 1 ml collagen protein solution were then mixed in a 35 mm × 10 mm culture dish. The dish was incubated horizontally for 10 min at room temperature. After the gel formed, 1 ml Dulbecco’s Modified Eagle’s Medium (DMEM, Gibco, USA) was added. The lid was then removed, and the prepared PU membrane was mounted onto the dish. The PU membrane and dish were sealed with Teflon tape at the edge and weighed. The assembled dish was then placed into a 37 °C-incubator at 50% relative humidity. Meanwhile, 1 ml culture medium was weighed. After being cultured for 12 h, the assembled dish was weighed, and the fibroblast/collagen gel was then photographed. The residue of culture medium was calculated as follows:


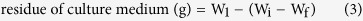


where W_1_ represents the weight of 1 ml culture medium and W_i_ and W_f_ represent the weights of the initial and the final assembled dish, respectively.

The final area of the gel was measured with IPP 6.0 software, and the rate of contraction was calculated using the following formula:


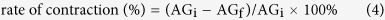


where AG_i_ represents the area of the initial gel (9.6 cm^2^) and AG_f_ represents the area of the final gel. This model is also referred to as the fibroblast populated collagen lattice (FPCL) model.

Subsequently, the fibroblasts/collagen gel was washed with PBS three times and then minced. The gel pieces were then digested with 1 ml 2.5 mg/ml trypsin (Boster, China). The digestion was terminated by adding 2 ml DMEM culture medium containing 10% fetal bovine serum (FBS, Gibco, USA) after incubating at 37 °C for 10 min. The number of cells was then counted using a hemocytometer (ReaCon, China).

To observe the effect of moisture regulated by PU membranes with different WVTRs on epidermal cell proliferation, HaCat cells were cultured using the above three-dimensional model. The HaCat cell suspension was adjusted to 1 × 10^4 ^cells/ml. Then, 700 μL cell suspension, 100 μL 10× PBS and 1 ml collagen protein solution were mixed in a 35 mm × 10 mm culture dish. Three ml Roswell Park Memorial Institute-1640 (RPMI-1640, Gibco, USA) culture medium was subsequently added to the dish after the gel formed. The other procedures used to establish the model were the same as those described above. The assembled dish was weighed, and the cells were cultured at 37 °C in a 5% CO_2_ incubator. After 36 h, this assembled dish was weighed again, and the residue of culture medium was calculated as described above. The HaCat cells were then digested from the gel using trypsin, and the number of cells was counted by a hemocytometer.

### Survey of the wound healing process

The dorsal surfaces of the Balb/c mice were shaved two days before the experiment. The mice were placed under anesthesia using 1% pentobarbital (Sigma, USA) via intraperitoneal injection (5–10 μl/g of body weight). After disinfecting with 75% alcohol, a 10 mm × 10 mm full-thickness wound was prepared by excision on the back of the mouse. A piece of sterilized 13 × 13 mm prepared PU membrane was sutured to the wound using 6.0 nylon. Wounds without any treatment were considered to be the blank group. On days 0, 1, 3, 5 and 7 postsurgery, the wounds were photographed. The initial or left areas of the wounds were measured using the IPP 6.0 software based on the pictures taken previously, and the rate of wound healing was calculated using the following formula:


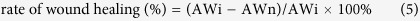


where AWi represents the area of the initial wound and AWn is the area of the wound on the nth day postsurgery^9^.

### Evaluation of wound contraction

The contracted area of each wound was measured and calculated as previously described^29^, and the rate of wound contraction was calculated as follows:


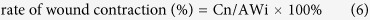


where Cn represents the contracted area on the nth day.

### Analysis of the neo-epithelium and granulation tissue during wound healing

On days 3 and 7, mice were sacrificed, and the wound tissues were carefully biopsied. Harvested wound tissues were divided into two equal parts for the following experiments (histological observation or western blot assay). Tissues were fixed with 4% formaldehyde, embedded in paraffin, and sectioned at a thickness of 5 μm. Hematoxylin and eosin (HE) staining and histological analysis were performed as described previously^9^. The length of the neo-epithelium and the granulation thickness were determined using the IPP 6.0 software, and the measurement procedures were performed blindly by two pathologists. Fifteen sections from five mice of each group at each time-point were analyzed.

### Immunohistochemistry and immunofluorescence

To investigate the key factors involved in wound contraction and keratinocyte proliferation in the wound tissue, α-SMA and PCNA were detected using immunohistochemical staining.^20^,^21^,^30^. The paraffin sections were deparaffinized and rehydrated. After incubation in a 99 °C water bath for 15 min, the sections were incubated with 3% H_2_O_2_ for 15 min. Sections were then blocked with 10% normal goat serum (Zhongshan Biology Company, China) for 1 h at 37 °C, which was followed by incubation with primary antibody (anti-α-SMA antibody ab5694, 1:500 dilution, Abcam, UK; anti-PCNA antibody ab15497, 1:1000 dilution, Abcam, UK) overnight at 4 °C. Sections were incubated with biotinylated goat-anti-rabbit IgG antibody (Zhongshan Biology Company, China) for 15 min at 37 °C and sequentially incubated with avidin peroxidase reagent (Zhongshan Biology Company, China). Diaminobenzidine solution was used as the chromogenic agent. After counterstaining with Hematoxylin, sections were photographed using an optical microscope (CTR6000, Leica, Germany).

To investigate keratinocyte migration at the wound edge, expression of E-cadherin was detected by immunofluorescence^15^. At day 3 postsurgery, wound tissues were collected and fixed with 4% formaldehyde, and they then underwent graded sucrose dehydration. Tissues were frozen in liquid nitrogen and sectioned at a thickness of 5 μm. The cryosections were balanced at room temperature for 15 min, blocked with 10% normal goat serum for 1 h and then incubated with anti-E-cadherin antibody (sc-7870, 1:200 dilution, Santa Cruz Biotechnology, USA) overnight at 4 °C. Sequentially, sections were incubated with Cy3-labeled secondary antibody (1:100, Boster, China) for 1 h at 37 °C. Finally, sections were counterstained with 4′,6-diamidino-2-phenylindole (DAPI, Beyotime, China) and observed using a Leica fluorescent microscope (CTR6000, Leica, Germany).

### Determining the expressions of PCNA and E-cadherin in the epidermis and α-SMA in wounds using western blot assays

Wound tissues were harvested as mentioned in the section above. To detect the expression of PCNA and E-cadherin in the epidermis, wound edge tissue was incubated in 5 mg/ml Dispase II (Sigma, USA) for 1 h at 37 °C, and then the epidermis was separated. Samples were weighed, frozen and minced in liquid nitrogen, and then, sequentially, lysis buffer (KeyGEN, China) was added. After shaking for 15 min at 4 °C, homogenates were centrifuged at 14000 rpm for 15 min; the supernatants were then collected. Protein concentrations were determined by the bicinchoninic acid (BCA) method in accordance with the manufacturer’s instructions (Thermal Scientific, USA). Equal amounts of protein were mixed with reducing sodium dodecyl sulfate (SDS) sample buffer and boiled for 5 minutes before loading the samples onto 10% SDS-PAGE gels. Thirty micrograms of protein were used for western blot analysis. Electrophoresis was performed at 80 volts for 0.5 h and then at 100 volts for 1.5 hours. The proteins were then transferred to a nitrocellulose (NC) membrane (GE, USA) at 100 volts for 1.5 hours. The NC membrane was blocked with tris-buffered saline (TBS) containing 5% bovine serum albumin (BSA, Biosharp, China) for 3 hours at room temperature, and the membrane was incubated with primary antibody (anti-PCNA antibody ab15497, 1:500 dilution, Abcam, UK; anti-E-cadherin, sc-7870, 1:500 dilution, Santa Cruz Biotechnology, USA; anti-β-actin was used as an internal control, 1:2000 dilution, Sungene, China) at 4 °C overnight. The membrane were subsequently washed with TBS containing 1% Tween-20 5 times followed by incubation with HRP-labeled goat anti-rabbit secondary antibody (1:2000) (Zhongshan Biology Company, China) for 1 h at room temperature. The NC membrane was washed with TBS containing 1% Tween 20 5 times and then visualized using enhanced chemiluminescence (Thermal Scientific, USA)^26^.

To detect the expression of α-SMA (anti-α-SMA antibody ab5694, 1:2000 dilution, Abcam, UK), wound tissues (including tissue from the epidermis, dermis and granulation tissue) were directly frozen and minced in liquid nitrogen. The other procedures were the same as mentioned above.

### Assaying the concentration of EGF in the wound exudates

The dorsal surface of the mice was first shaved and disinfected, followed by the preparation of a 10 mm × 10 mm full-thickness wound by excision on the back of the mouse. Two sterilized polyurethane tubes (10-mm length, 1.02-mm inner diameter and 1.65-mm outer diameter) were implanted in each mouse as previously reported^31^. Then, a piece of sterilized 13 × 13 mm prepared PU membrane was sutured and dressed on each wound. Three days after implantation, the fluid samples inside the tubes were collected for cytokine analysis. The exudate samples were diluted with sample diluents (R&D Systems, USA) at 1:200 dilution. Then, the samples were measured with the Mouse EGF Elisa Kit (R&D Systems, USA) in accordance with the manufacturer’s instructions. Briefly, 100 μL of standard or sample was added to each well. After incubating for 90 min at 37 °C, each well was washed 5 times with wash buffer. A 100-μL aliquot of mouse EGF conjugate was subsequently added to each well and incubated at 37 °C for 2 h. Subsequently, each well was washed again, and 100 μL of substrate solution was added, followed by the addition of 100 μL of stop solution after incubation for 30 min. The optical density was measured at 450 nm using an enzyme-linked immunosorbent assay reader (Thermo Varioskan Flash, USA).

### Statistical analysis

Statistical comparisons were performed using a one-way ANOVA test, followed by Bonferroni’s test. All data are presented as the mean ± standard deviation (SD). p values less than 0.05 were considered significant.

## Additional Information

**How to cite this article**: Xu, R. *et al*. Controlled water vapor transmission rate promotes wound-healing via wound re-epithelialization and contraction enhancement. *Sci. Rep*. **6**, 24596; doi: 10.1038/srep24596 (2016).

## Supplementary Material

Supplementary Information
