# Supplementary material for: Controlled water vapor transmission rate promotes wound-healing via wound re-epithelialization and contraction enhancement
Source: Sci Rep. 2016 Apr 18;6:24596. doi: 10.1038/srep24596 (PMC4834567; doi:10.1038/srep24596)
Supplement: Supplementary Information [file srep24596-s1.doc]

**Supplementary information**

**Controlled water vapor transmission rate promotes wound-healing via wound re-epithelialization and contraction enhancement**

Rui Xu^1^, Hesheng Xia^2^, Weifeng He^1^, Zhichao Li^2^, Jian Zhao^2^, Bo Liu^2^, Yuzhen Wang^1^, Qiang Lei^1^, Yi Kong^1^, Yang Bai^1^, Zhihui Yao^1^, Rongshuai Yan^1^, Haisheng Li^1^, Rixing Zhan^1^, Sisi Yang^1^, Gaoxing Luo^1,*^, Jun Wu^1,*^

^1^ Institute of Burn Research, Southwest Hospital; State Key Lab of Trauma, Burn and Combined Injury; Chongqing Key Laboratory for Disease Proteomics, Third Military Medical University, Chongqing 400038, China.

^2^ State Key Laboratory of Polymer Materials Engineering, Polymer Research Institute of Sichuan University, Chengdu 610065, China.

* Corresponding authors: Gaoxing Luo, MD, PhD & Jun Wu, MD.

E-mail: logxw@yahoo.com (Gaoxing Luo)

[editorinchief@burninchina.com](mailto:editorinchief@burninchina.com) (Jun Wu)

Address: Institute of Burn Research, Southwest Hospital, Third Military Medical University, Chongqing 400038, China.

Tel: 0086-23-68752688, Fax: 0086-23-68752688


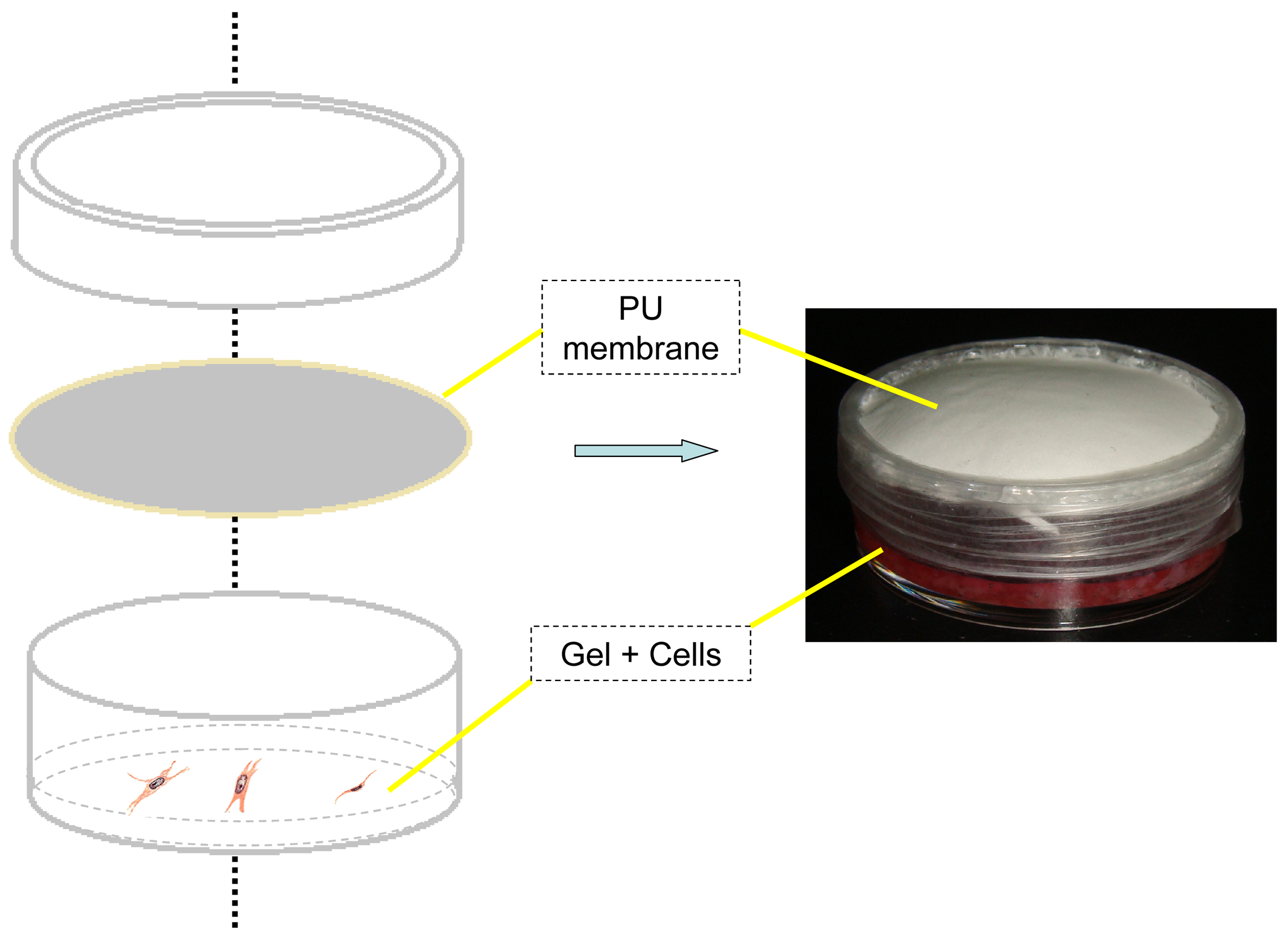


Figure S1. Establishment of the three-dimensional culture model. To observe the effects of moisture regulated by PU membranes with different WVTRs on proliferation and function of fibroblasts and epidermal cells, an in vitro three-dimensional culture model was established.


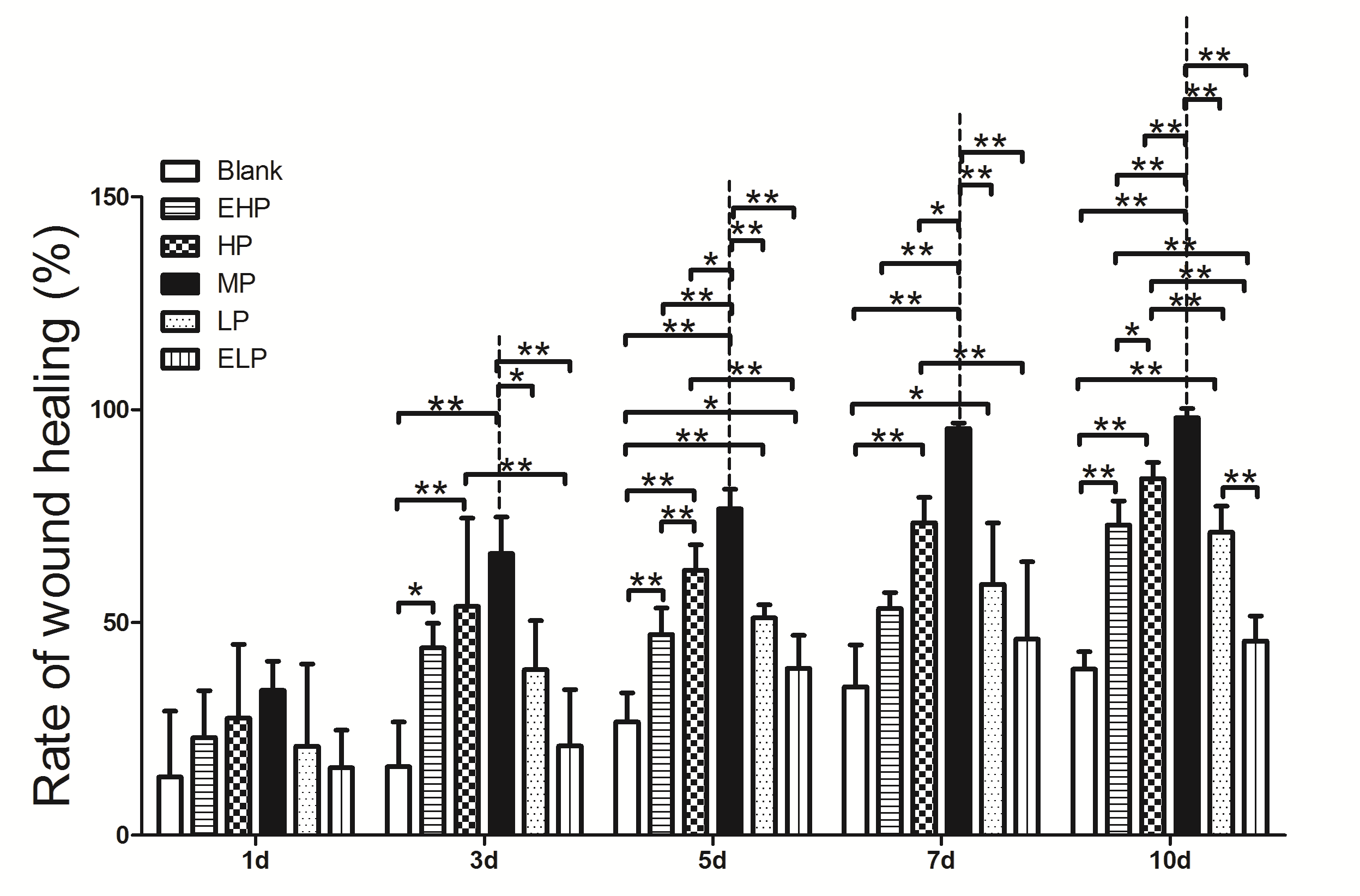


Figure S2. The rates of wound healing at different times. The values were calculated as the mean ± SD (n = 5), **p < 0.01, *p < 0.05.

**
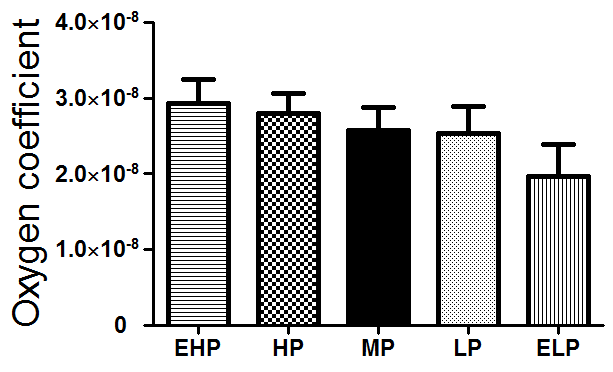
**

Figure S3. Oxygen coefficient of different membranes. The values were calculated as the mean ± SD (n = 3)
